# Impacts of climate warming on reindeer herding require new land-use strategies

**DOI:** 10.1007/s13280-021-01655-2

**Published:** 2021-12-17

**Authors:** Gunhild C. Rosqvist, Niila Inga, Pia Eriksson

**Affiliations:** 1 Department of Physical Geography, Stockholm University, 106 91 Stockholm, Sweden; 2 Krokvik, 17, 981 95 Kiruna, Sweden

**Keywords:** Climate warming, Indigenous knowledge, Land use, Northern Sweden, Reindeer herding, Snow conditions

## Abstract

Climate in the Arctic has warmed at a more rapid pace than the global average over the past few decades leading to weather, snow, and ice situations previously unencountered. Reindeer herding is one of the primary livelihoods for Indigenous peoples throughout the Arctic. To understand how the new climate state forces societal adaptation, including new management strategies and needs for preserved, interconnected, undisturbed grazing areas, we coupled changes in temperature, precipitation, and snow depth recorded by automatic weather stations to herder observations of reindeer behaviour in grazing areas of the Laevas Sámi reindeer herding community, northern Sweden. Results show that weather and snow conditions strongly determine grazing opportunities and therefore reindeer response. We conclude that together with the cumulative effects of increased pressures from alternative land use activities, the non-predictable environmental conditions that are uniquely part of the warming climate seriously challenge future reindeer herding in northern Sweden.

## Introduction

Reindeer (*Rangifer tarandus)* herding is one of the primary livelihoods for Indigenous peoples throughout the Arctic. High resilience to stress caused by environmental and anthropogenic factors has characterized historical reindeer herding (Brännlund and Axelsson 2011). For many communities’ cumulative impacts from natural resource exploitation, tourism and a rapidly changing climate now pose serious challenges to reindeer herding (Tyler et al. 2007; Eira et al. 2018) such as in northern Sweden. Although the Sámi here have experienced that multitude of societal pressures including mining, forestry, hydropower, wind power, and tourism has limited their herding practices over the past 130 years or so (Moen 2008; Berg et al. 2009; Horstkotte et al. 2014; Klocker-Larsen et al. 2017; Skarin et al. 2018), the climate warming pressure is perceived to be the most imminent pressure and overshadows other pressures by having large spatial coverage. Still the effect of warming on reindeer land use is poorly understood. Here, we present a joint effort between scientists and reindeer herders to assess the impact of recent climate warming on reindeer land use in northern Sweden.

**Fig. 1:**
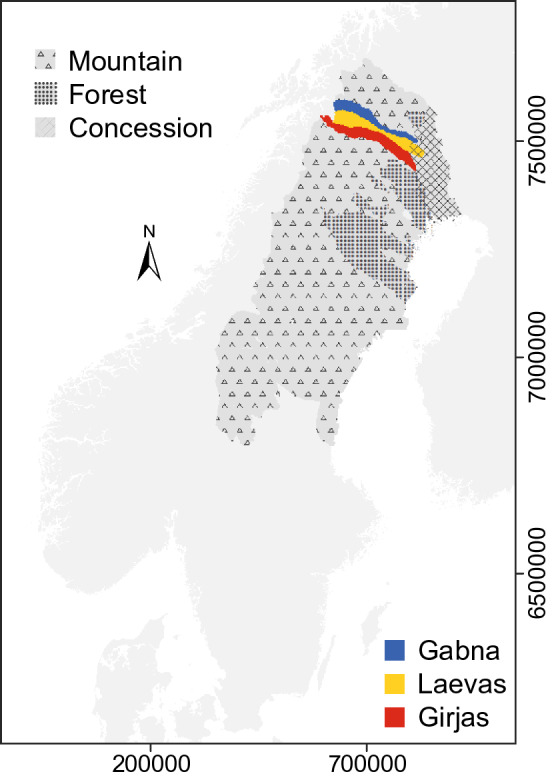
The Sámi reindeer herding area of Sweden is shown in darker gray color. Hatched areas mark the extensions of the three different categories of Sámi reindeer herding communities, i.e., mountain, forest, and concession communities (Länsstyrelsernas geodatakatalog 2020). The colored areas show the grazing areas used by the Gabna, Laevas, and Girjas reindeer herding communities which all are ‘mountain’ communities

Reindeer herding in Sweden is practiced by 51 Sámi Reindeer Herding Communities (RHCs) over c. 160 000 km^2^ (Fig. 1). The current division of the land between RHCs, the number of reindeer allowed per RHC, and their seasonal land use are regulated by The Reindeer Husbandry Act (Sveriges Riksdag 1971). State regulations have impacted Sámi reindeer herding in Sweden since the end of the nineteenth century and limited their land-use flexibility (e.g., Löf 2013; Brännström 2017; Grönvall and Löf 2020). Prior to the commencement of resource exploitation in northern Sweden, the semi-domesticated reindeer could migrate over large distances between winter pastures in the eastern forested lowlands and summer pastures in the western mountains, and between pastures within seasons. Such practice allows reindeer to adapt to the large seasonal changes in weather and to variable snow conditions. Gatherings for reindeer round-ups for inventory and marking, and migration have traditionally been flexible because they are timed after the animal’s natural response to grazing conditions.

Weather is an important driver of reindeer migration as they search for grazing all year (e.g., Forbes and Kumpula 2009; Horstkotte et al. 2017). The most critical weather periods are during the winter and the spring (e.g., Albon et al. 2017). The amount and quality (i.e., density, hardness, and wetness) of the snow are key in determining reindeer access to terricolous (ground) and arboreal (tree) lichens during winter (Skogland 1978; Roturier and Roué 2009; Callaghan et al. 2011; Kivinen and Rasmus 2014; Forbes et al. 2016; Turunen et al. 2016). A dry and frozen ground at first snowfall provides the best conditions. If the ground remains unfrozen and moist, ice crystals often form on the ground and on the vegetation covering the ground, on which further build-up of ice easily occurs which impact the whole ecosystem (Kausrud et al. 2008; Bokhorst et al. 2016). Rain-on-snow events, followed by refreezing, often create hard and icy layers at the surface and in the snowpack (Bokhorst et al. 2016; Rasmus et al. 2018). Such conditions radically decrease the accessibility of and sometimes also even the ability to smell and identify terricolous lichens. Wet snow can stimulate the growth of mycotoxin-producing molds and toxic lichens (Kumpula et al. 2000). Large decreases in reindeer population in Arctic Russia (Forbes et al. 2016) and on Svalbard (Kohler and Aanes 2004) have previously been associated with icing on the ground and in the snowpack.

A critical period is around the time of calving, in mid-May, when female reindeer (vaja) grazing options and the survival rate of calves are determined by the weather, plant phenology (Tveraa et al. 2013; Chen et al. 2018), predation by wolverines, wolves, bears, and eagles, and human disturbance (Sivertsen et al. 2016; Mattisson et al. 2016). Plant phenology and the availability of nutrient-rich vegetation are strongly coupled to spring temperature and snow melt (Pettorellin et al. 2005; Mårell et al. 2006). A delayed access to such vegetation negatively influences reindeer health and therefore also the growth rate of calves (Paoli et al. 2020).

We have coupled changes in temperature, precipitation, and snow depth, recorded by automatic weather stations (AWS), to herder observations of reindeer behavior and migration in and between core grazing areas of the Laevas Sámi reindeer herding community, northern Sweden between 2013 and 2018. By combining indigenous knowledge with scientific measurements (Huntington et al. 2004), we were able to show how recent weather and snow changes impact reindeer behavior and herding strategies. The results show how essential preserved ecosystems and interconnected grazing areas are for adaptive strategies to succeed in a warmer climate with uncertain environmental consequences.

### Study area

**Fig. 2:**
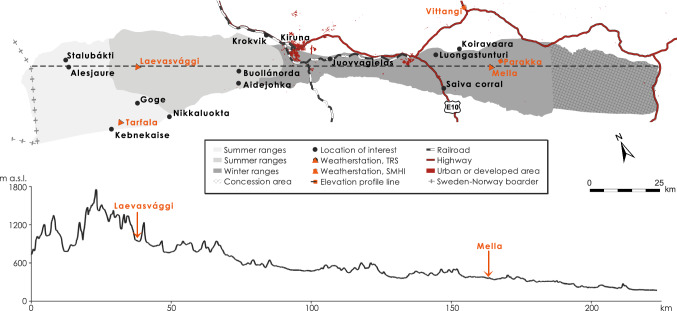
Map shows the extension of, and elevation profile for, Laevas reindeer herding community land use. Light gray color delimits area of summer ranges; middle gray color spring and autumn ranges, and dark gray color winter ranges. The hatched area in the east delimits an area used primarily by Tärendö concession Sámi community (Sveriges Riksdag 1971). Symbols and place names showing locations of interest are black, symbols and place names showing locations of weather station are orange

**Fig. 3:**
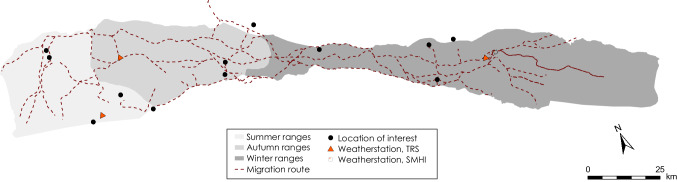
Map shows migration routes used by Laevas RHC

The grazing land used by Laevas Sámi RHC stretches from the mountainous Norwegian border in the west to the forested lowlands c. 80 km southeast of Kiruna (Figs. 1, 2). Traditionally the large-scale migration (from the east towards the west) between winter and spring pastures occurs in March–April, and between autumn and winter pastures (from the west towards the east) in November–December (Fig. 3). Before natural resource exploitation began with iron-ore mining at the end of the nineteenth century (Avango et al. 2019) and industrial forestry, it was possible to practice highly flexible herding in an interconnected landscape (e.g., Brännlund and Axelsson 2011) allowing for the seasonal ranges to be utilized in a sustainable manner. The prime calving and spring grazing ranges for Laevas RHC used to be the slopes of Kirunavaara and Luossavaara (where *vaara* is mountain) that are now partly removed by mining and the home of disruptive mining-related infrastructure such as the town of Kiruna (Sjöholm 2013). The current prime calving location is in Laevasvággi (where *vággi* is valley), located above the tree line at medium elevation in the Swedish mountains (Fig. 2). The tree line in the area occurs at c. 700 m above sea level (m a.s.l.) and consists of birch (*Betula pubescens ssp. czerepanovii* and *Betula nana*) (Fig. 2). The Rautas river forms the natural northern boundary of Laevas reindeer grazing lands (bordering Gabna RHC) and Kalix river the southern boundary (bordering Girjas RHC) (Fig. 1). Despite such natural borders, reindeer often cross over into areas administered by neighboring RHC. Summer pastures of the Laevas RHC are located within the Swedish mountains close to the Norwegian border (Fig. 2) and a summer camp has been established at the eastern shore of Alesjaure (where *jaure* is lake) (Fig. 2).

Reindeer mainly feed on graminoids, forbs, and leaves of shrubs (Mårell et al. 2006) in the summer and early autumn to build energy reserves for the next winter. Usually the reindeer movement is guided by topography. Due to the pronounced topography of the alpine mountains herding tends to be efficient as the reindeer stay closer together. In contrast, reindeer disperse over wider areas on the topographically more diffuse mountain plateaus.

If summer days are hot reindeer migrate high into the mountains, seeking relief from heat and insects on snowfields and glaciers (Skarin et al. 2010). In the high alpine valleys, they feed on the limited resources offered by fresh vegetation growing below snowfields. At these high elevations, where vegetation is too sparse to support a large number of animals, the reindeer risk a low overall nutrient intake. Herders have noted that during cooler periods in summer, or indeed during the night, reindeer tend to move down-valley several kilometers to graze nutrient-richer pastures. Even if warmth persists, the shorter days and cooler and more humid nights of the early autumn attract reindeer to even lower altitudes. They then start to track along and across the alpine valleys towards the east, where they like to feed on, for example, mushrooms. Because rainfall amounts decrease towards the east across the mountains (Fig. 2), fresh vegetation is not provided here to the same extent as further west. Further east again, the open plateau areas west of Kiruna are mainly used as transitional land during spring and autumn migrations between winter and summer pastures (Fig. 2, 3). While reindeer pause to graze in these uplands west of Kiruna, usually in mid-September, they are rounded up for slaughter at Aidejohka (Fig. 2).

The pine and spruce forest ecosystems offer grazing of terricolous and arboreal lichens in the traditional winter pastures located southeast of Kiruna (Fig. 2). Laevas RHC divide these winter pastures into eight winter groups called siidas. Siidas are groups based on family kinship and is an important self-governance tool that provides increased flexibility and more sustainable winter grazing (Vuolja-Magga 2012; Labba 2016). Herders gather their reindeer for identification and distribution into siidas usually at Buollánorda (Fig. 2). This community activity can only happen when a continuous snow cover is established and ice covers the rivers, mires, and lakes that constitute parts of traditional migration routes (Fig. 3). The timing, therefore, varies between years but usually gatherings occur sometime in November or December. Herders use snowmobiles to guide their reindeer along predetermined perceived safe routes to the winter grazing areas (Fig. 3). Autumn weather determines the ground conditions in the winter ranges at the time of first snowfall (frozen, thawed). Ground conditions and lichen accessibility are always assessed before the RHC decides how they best collectively and most optimally use the winter grazing area. During their daily patrolling, the herders continually monitor grazing conditions and animal health of their siida during winter. Herders determine the snow conditions by digging with their hand through the snowpack feeling if there is any stratigraphic difference in hardness. They also try to identify any evidence of mold on the ground by smelling. The confined forest areas offer more easily herding and monitoring conditions than the open lowland mountain area west of Kiruna because the animal footprints are not wind swept and the herders are less exposed to rough weather.

The reindeer herding year can be divided into eight seasons which are directly related to the traditional annual behavioral patterns of the *reindeer *(Manker 1975). The year begins with the spring season when the calves are born. Herders find that a large diurnal temperature range providing sunny and mild days and cold nights is optimal during the spring (April–May), hoping that a crust forms on the snowpack surface easing reindeer movement from the forests in the east to the calving lands. Dry weather keeping the newborn calves dry is important in May. Warm, but not too high temperatures, and some rain provide the best conditions for early greening and good grazing conditions during early (June) and high summer (June–July). Too high temperatures force the reindeer to high altitudes where food is scarcer. Late summer (August) weather is best if cool, with some rain, so that the vegetation in the lower mountains in the east does not dry out. Clear cool and dry weather will help dry up the ground before the first snowfall during the autumn (September–October). Cold and dry weather is also favorable for the reindeer during early winter (November–December) and full winter (January–March). A maximum accumulation of 50 cm of dry, powdery snow is best for reindeer access to lichens. If grazing conditions become really critical during winter, female reindeer may abort their calves to allow themselves to survive (Albon et al. 2017).

## Materials and methods

We have determined the impacts of seasonal and event-type weather changes on reindeer movements and herding strategies. Temperature, precipitation, and snow depth data recorded by AWS was used together with reindeer herder knowledge held by one of the co-authors concerning grazing conditions, reindeer movement, and behavior for 5 years (2013–2018). For the reconstruction, we also used information about herding logistics, such as times and locations of gatherings and round-ups derived from daily diary notes, and detailed information of reindeer movements provided by GPS collars.

We analyze conditions relevant for the entire Laevas RHC reindeer herd of c. 8000 animals for the late spring/summer/early autumn period, when the herd roam free in the mountains. We merely analyze the conditions for reindeer belonging to the Inga family siida (c. 1000 animals) during the winters.

### Weather and snow data

As part of this study we have employed two AWS (Swedish Infrastructure for Ecosystem Science 2021) in core reindeer grazing areas of Laevas RHC (Fig. 1). We use weather station data of daily average, minimum, and maximum air temperature, snow depth, and rainfall after snowpack establishment because these parameters are known to directly or indirectly affect reindeer behavior. The daily snow depth recordings from the AWS Laevasvággi and AWS Mella concern maximum snow depth. The statistical relationships, i.e., the quadratic polynomial equation, between data from these two stations, and data from nearby weather stations managed by Tarfala Research Station and the Swedish Meteorological and Hydrological Institute (SMHI) were determined and implemented to fill gaps of missing data in the original datasets (Fig. 2, Table 1). Gaps in the temperature data from AWS Laevasvággi were filled with data from the weather station located at Tarfala Research Station. Gaps in the temperature data from AWS Mella were filled with data from the nearby weather station in Vittangi (SMHI 2020), and gaps in snow depth data were filled with data from Parakka (SMHI 2020) (Fig. 2, Table 1). Because rainfall during the snow cover period potentially promotes ice layer formation on or in the snowpack, such events were identified in the record of precipitation type and snow depth data from Parakka (SMHI 2020). Precipitation type (e.g., snow, rain) is based on manual observations recorded as a Swedish descriptive term for the event (SMHI 2020). We selected events when precipitation was classified as sleet squall, sleet, rain, or rain shower because we consider these types to likely affect the snowpack conditions. In contrast precipitation classified as snow, snow squall, graupel, soft hail, hail, and drizzle were considered not to considerably promote building of ice layers on or in the snowpack and were, therefore, not included in the analysis.

**Table 1:** Name and location of automatic weather station from which values of daily average, minimum, and maximum air temperature (Temp), snow depth (SD), and precipitation amount and type (Prec) were used to the diagrams displayed in Figs. 4, 6, 8, 10, and 12

Location	Parameter	Coordinates (SWEREF99 TM)	Altitude (m a.s.l.)	Time interval (yyyy-mm-dd)
Laevasvággi	Temp, SD	N 7552960; E 665289	953	2014-05-10 2014-10-10
				2015-05-10 2015-10-10
				2016-05-10 2016-10-10
				2017-05-10 2017-10-10
				2017-10-01 2018-02-06
				2018-02-28 2018-04-31
				2018-05-10 2018-10-10
Mella	Temp, SD	N 7500636; E 780440	355	2014-11-12 2015-04-31
				2015-10-01 2016-04-31
				2016-10-01 2017-04-31
Parakka	Prec	N 7500921; E 784496	340	2013-10-01 2014-04-31
				2014-10-01 2015-04-31
				2015-10-01 2016-04-31
				2016-10-01 2017-04-31
Vittangi	Temp	N 7523827; E 780519	250	2013-10-01 2014-04-31
				2014-10-01 2014-11-11
Parakka	SD	N 7500921; E 784496	340	2013-10-01 2014-04-31
				2014-10-01 2014-11-11
Tarfala	Temp	N 7537421.8; E 651517.5	1143	2018-02-07 2018-02-27

### Reindeer movements

Information about habitat use is based on herder knowledge of the whereabouts of the reindeer. The reindeer are herded between seasonal pastures by snow mobiles in early winter and early spring and the perimeter of the area designated to the siida is patrolled daily in winter. ATVs and sometimes helicopters are used to gather reindeer for round-ups in the mountains during the summer and autumn. To aid herd management, Laevas RHC has tracked reindeer using GPS collars since 2013 (Tannak reindeer tracking network system; Sjödin, 2017). This method allows the herd to be monitored remotely and provides detailed information of reindeer movements (Skarin et al. 2010). The GPS positions, which were recorded once a day, were fed into a GIS system (RenGIS) which is used by Swedish RHCs to trace and describe their land use (Jougda and Kemi 2014; Jougda 2020). To provide the most representative picture of how the reindeer use their ranges, the collared animals were carefully selected on the basis of their habit to remain on the RHC ranges and strength to carry the collar. In total c. 800 (10%) reindeer of the full RHC herd, and 70 reindeer from the Inga family siida were collared. Information about herding logistics such as times and locations of gatherings and round-ups was derived from daily diary notes written by Agneta Inga (A. Inga, pers. comm.).

## Results

Based on data recorded by automatic weather stations, GPS positions of reindeer, herder observations in the field, and information provided in a herder diary (A. Inga, pers. comm.), we reconstructed weather, snow grazing conditions, and herd movements for the period between November 2013 and October 2018 (Tables 1, 2, 3, and 4; Figs. 4, 5, 6, 7, 8, 9, 10, 11, 12, and 13).

**Table 2:** The timing of rain-on-snow events for the period displayed in the diagrams (Figs. 4, 6, 8, 10, and 12) during the study period 2013–2018 at Parakka

Winter 2014	Winter 2015	Winter 2016	Winter 2017	Winter 2018*
2013-10-22	2014-11-10	2015-11-09	2016-12-15	2017-10-28
2013-12-10	2015-03-01	2015-12-05	2017-01-18	2017-12-19
2013-12-28		2015-12-09	2017-01-26	
2013-12-29				
2014-01-02				
2014-01-03				
2014-02-09				
2014-02-10				
2014-02-17				
2014-02-24				
2014-02-27				
2014-03-07				

*Reindeer not in the winter ranges

**Table 3:** Average monthly air temperature (°C) measured at Laevasvággi and Mella

Location, Season	Oct	Nov	Dec	Jan	Feb	Mar	Apr	May	Jun	Jul	Aug	Sep
Mella, 2014 Laevasvággi, 2014	− 1.6	− 8.8	− 9.4	− 17.8	− 4.2	− 4.2	− 0.3	0.0	6.1	15.0	9.4	4.7
Mella, 2015 Laevasvággi, 2015	− 0.7	− 8.6	− 9.7	− 13.8	− 7.1	− 3.3	0.3	− 0.2	3.6	8.5	10.8	6.6
Mella, 2016 Laevasvággi, 2016	0.3	− 4.7	− 8.1	− 17.6	− 9.6	− 5.0	− 0.6	2.0	5.7	11.2	7.3	6.1
Mella, 2017 Laevasvággi, 2017	0.6	− 8.3	− 7.7	− 9.6	− 10.2	− 6.3	− 3.0	− 2.2	5.7	9.1	7.2	4.5
Mella, 2018 Laevasvággi, 2018	− 0.2	− 8.1	− 12.2	− 14.5	− 14.8	− 10.2	− 0.2	3.4	4.2	14.6	8.3	3.7

**Table 4:** The snow-free period in Laevasvággi and date when the snowpack starts to build up at Mella during the study period 2013–2018

Snow-free period	First snow
05 Jun–07 Oct, 2014	20 Nov, 2014
05 Jul–04 Nov, 2015	06 Nov, 2015
01 Jun–26 Oct, 2016	05 Nov, 2016
15 Jun–02 Oct, 2017	26 Oct, 2017
22 May–21 Oct, 2018*	02 Dec, 2018

*Ground snow covered between 26 Sep and 13 Oct

**Fig. 4:**
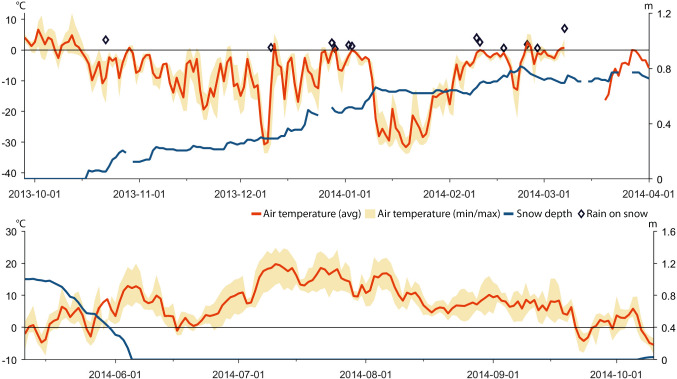
Diagrams display daily average and min/max air temperatures (left *X*-axis), snow depth (right *X*-axis), and rain-on-snow events for the herding season 2013–2014

**Fig. 5:**
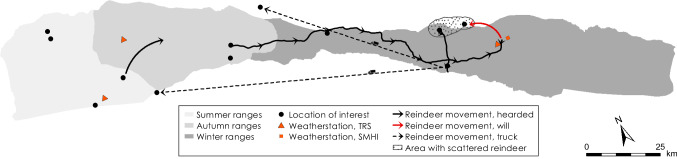
Logistics for the herding season 2013–2014

**Fig. 6:**
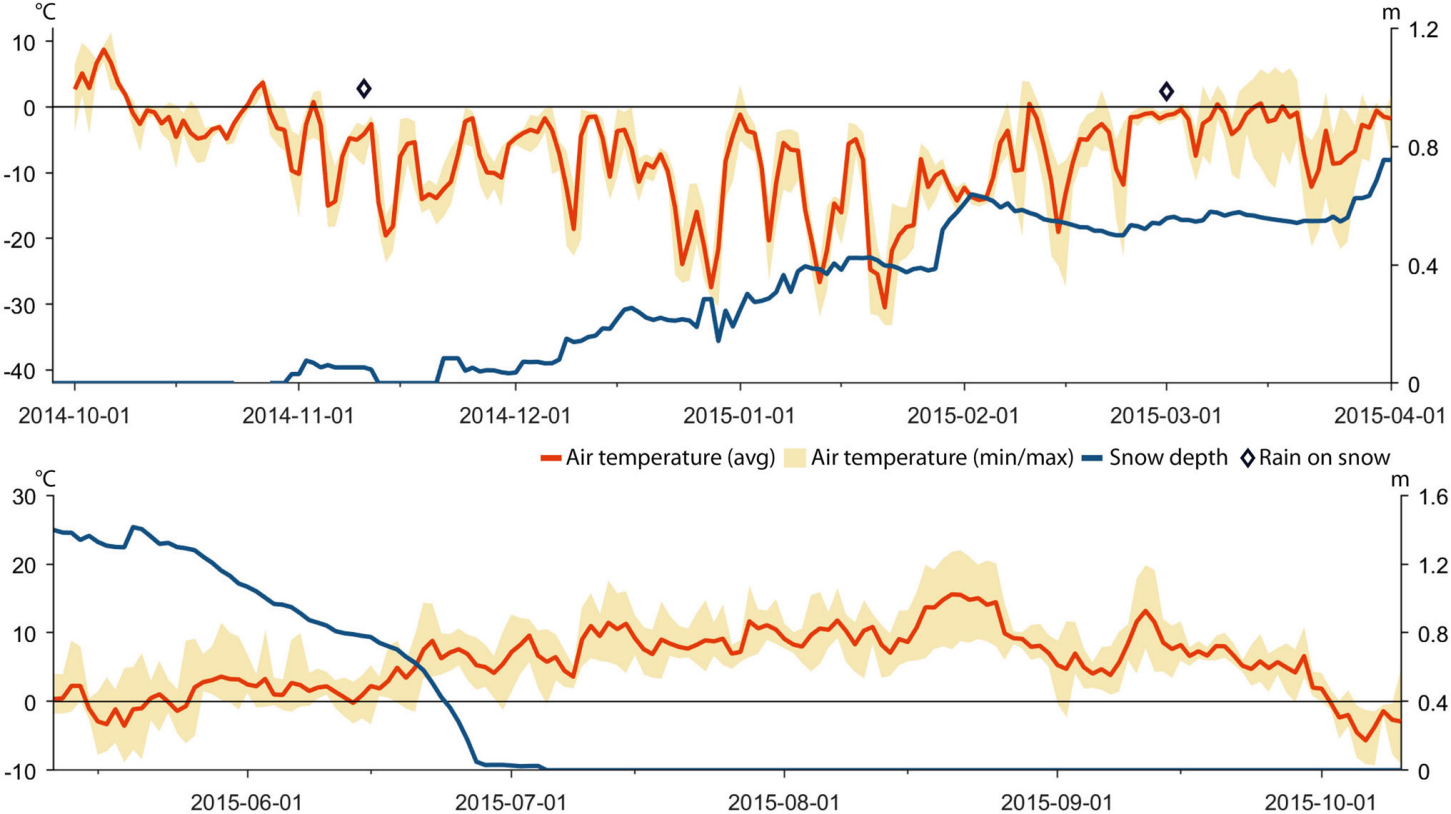
Diagrams display daily average and min/max air temperature, snow depth, and rain-on-snow events for the herding season 2014–2015

**Fig. 7:**
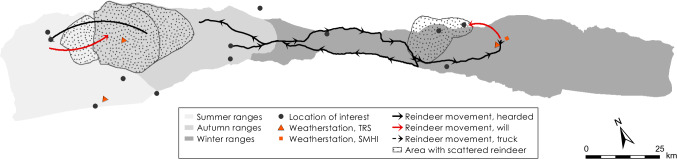
Logistics during the herding season 2014–2015

**Fig. 8:**
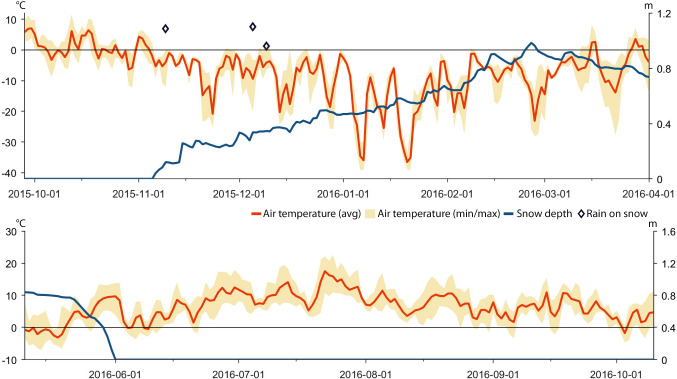
Diagrams display daily average and min/max air temperatures, snow depth, and rain-on-snow events for the herding season 2015–2016

**Fig. 9:**
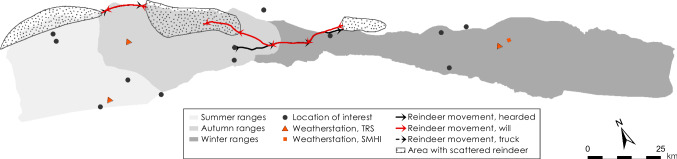
Logistics during the herding season 2015–2016

**Fig. 10:**
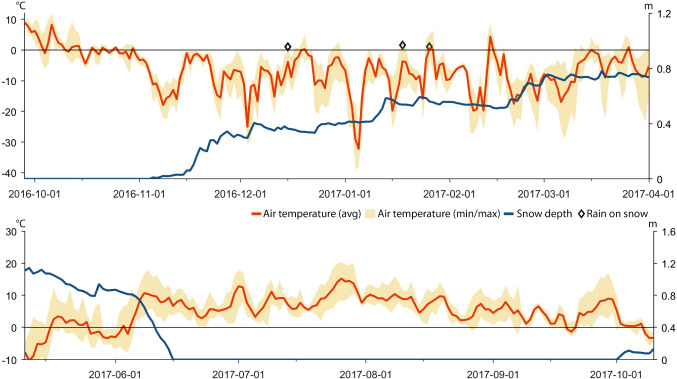
Diagrams display daily average and min/max air temperatures, snow depth, and rain-on-snow events for the herding season 2016–2017

**Fig. 11:**
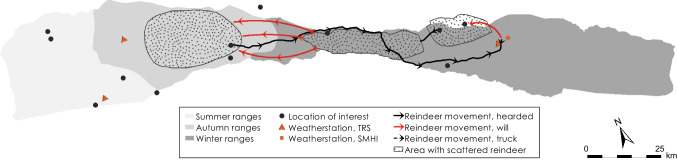
Logistics during the herding season 2016–2017

**Fig. 12:**
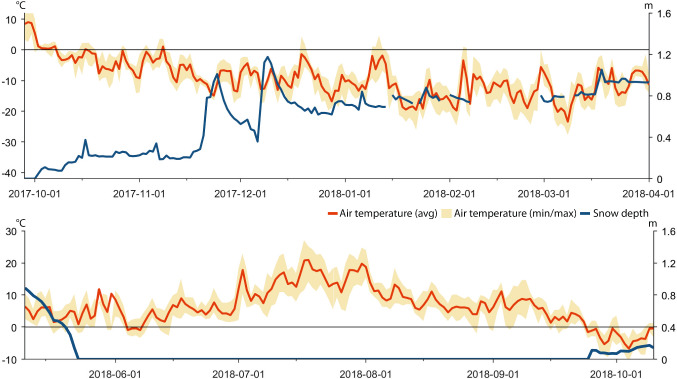
Diagrams display daily average and min/max air temperatures, snow depth, and rain-on-snow events for the herding season 2017–2018. Data from the weather station located in Laevasvággi represent all seasons because the reindeer did not graze in the winter ranges

**Fig. 13:**
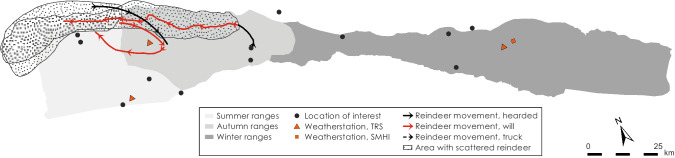
Logistics during herding season 2017–2018

### Herding season 2013–2014

The full reindeer herd was gathered into round-ups for separation into siidas at Buollánorda (Fig. 2) in late November 2013. In early December, the Inga family siida were herded eastward to the pine forest around Mella in early December (Figs. 2, 5) despite uncertain grazing conditions. Here, the herders note that reindeer had difficulties to access terricolous lichens. These observations agree with the weather data recorded by AWS Mella for this period, which indicate that several rain-on-snow events occurred in December and early January (Table 2; Fig. 4) and that the temperature suddenly dropped in early January (Fig. 4) further hardening the snowpack and also icing up the arboreal lichens. Herder observations and GPS data show that the reindeer moved and spread to graze arboreal lichens in the mixed forest at Koiravaara following this period (Figs. 2, 5). Because the conditions for the reindeer became untenable, the decision was made to gather as many animals as possible from the scattered herd and guide them via Luongastunturi to the Saiva corral (Figs. 2, 5). From here, the majority of the reindeer were transported by truck to Nikkaluokta, a distance of c. 120 km, and then herded to the Goge mountain area (Figs. 2, 5). Some of the weakest calves were transported to a corral located 10 km northwest of Kiruna, where they were fed with silage and pellets during the rest of the winter. Grazing conditions at the Goge mountain were good because terricolous lichens were exposed on snow-free rocks. The reindeer were herded to Laevasvággi in early May for calving. The GPS data show that some of the reindeer that were left behind followed traditional migration routes westward in the spring. The weather and snow data reveal that temperatures remained low and the valley did, therefore, not become snow free until early June. In contrast, July was uncommonly warm. The average July 2014 temperature in Laevasvággi was 15.0 °C (Table 3), which can be compared to the 5-year average for July of 11.7 °C. By July, the reindeer grazed the mountains bordering Norway west of Alesjaure (Fig. 2). To spare the reindeer who were suffering from the heat, the herders reduced the number of round-ups for calf labeling which limited their opportunities to associate newborn calves to a particular vaja and siida. The reindeer were left grazing undisturbed throughout August, when the AWSs record show quite normal temperatures (Table 3).

### Herding season 2014–2015

Herders waited as long as possible in the autumn 2014 before deciding which locations to use for winter grazing. The temperature record of November (Fig. 6) indicated a high risk for icing of the ground. The GPS data show that the reindeer started to migrate westward towards the mountains in early December which is contrary to planning. Herders then hurried to gather the reindeer, separate them into siidas, and guide them to the pine forest at Mella. Here, grazing conditions turned out to be much worse than anticipated (A. Inga, pers. comm.). The ice layer that had formed at the bottom of the snowpack, preventing reindeer to access terricolous lichens, was most likely the result of the rain-on-snow event that was recorded by AWS Mella in early November (Fig. 6). The GPS data show that as a result, the reindeer themselves headed to the Koiravaara area where the snow on the ground and in the trees was less icy (Figs. 2, 7).

The GPS data reveal that the massive snowfall event of approximately 30 cm at the end of January 2015, recorded by AWS Mella (Fig. 6), completely halted reindeer migration and, therefore, also inhibited further grazing. The herders had to supplementary feed the reindeer (in total c. 2500 animals for the RHC) with silage during one month to prevent them from starving. Herders note that conditions were so strenuous that many vajas aborted their calves (A. Inga, pers. comm.). The snowpack had become hard enough to walk upon for the reindeer in the second half of February, allowing the reindeer, as confirmed by the GPS data, to migrate and spread out to arboreal lichens elsewhere (Fig. 7). The wind had scoured arboreal lichens from the trees which provided easily accessible food on top of the snowpack. The weather record shows large diurnal temperature variations during March, including above freezing (Fig. 6). This sustained the hard snow cover until April and eased the westward migration towards the calving lands in Laevasvággi. The GPS data show that the reindeer that were left behind because of deteriorated health migrated themselves westward towards the mountains during the summer. Tragically, many followed the railway embankment instead of the traditional route across the, at this time of year, wet mires, and c. 60 animals were killed (A. Inga, pers. comm.).

The weather data from AWS Laevasvággi show that temperatures were low most of June and a deep but generally declining snowpack, delayed the start of the vegetation season until early July (Fig. 6, Table 4). Hoping for good weather and grazing conditions further west, a large effort was made by the herders to gather the reindeer and herd them to the summer grazing lands (Fig. 7). However, July temperatures remained low (Fig. 6, Table 3), and the reindeer, therefore, only spent a short period of time in the western mountains before heading back east.

### Herding season 2015–2016

Weather data show that temperatures were relatively high during the autumn and early winter of 2015 and that several rain-on-snow events occurred after the snowpack formed in early November (Fig. 8). When the herders inspected the grazing conditions around Mella they noted that the terricolous lichens were covered in ice already early in the season. They postponed the decision of where to herd the reindeer for winter grazing until the siida separation on December 24. Then the Inga family eventually decided to herd the reindeer to Juovvagielas, which is located a relatively short distance east of Kiruna (Figs. 2, 9). Unfortunately ice crystals had formed on terricolous lichens here as well and after only 2 weeks, the reindeer responded by dispersing in search for accessible alternative pastures. The siida were herded back to west of Kiruna once more where the snowpack was less dense and therefore provided access to food. Reindeer positions provided by the GPS show that some reindeer continued further west all the way to the summer grazing areas around Alesjaure. These were gathered and kept at Stalubákti (Fig. 2) and were guided back to the calving lands in Laevasvággi at the end of April for optimal grazing. The snow depth data from AWS Laevasvággi show that the ground was snow free in early June providing good grazing conditions after calving (Fig. 8). The GPS positions show that the reindeer remained grazing at low elevation in the western mountains until late July which eased gathering for round-ups and effective labeling of the many surviving calves.

### Herding season 2016–2017

The reindeer were herded to an area southwest of Mella in early January 2017 (Fig. 11). The weather data from AWS Mella show that some events with positive air temperatures and rain-on-snow occurred in December and January (Fig. 10). The herders note that these events iced up the snowpack and that melting snow from the trees refroze on top of the snow, which further reduced the area with accessible terricolous lichens. In search for arboreal lichens, the reindeer responded by moving to Koiravaara. In early March, the reindeer were herded, to an area west of highway E10 (Figs. 2, 11) where they were allowed to spread out and, therefore, survive on arboreal lichens the rest of the winter. Tracking their movement using the information provided by the GPS collars, the herders let the reindeer continue westwards themselves towards Laevasvággi for calving in April. Here, the AWS recorded low temperatures in May and early June and a remaining snowpack until mid-June delaying reindeer access to forage (Fig. 10). In contrast, cool July temperatures (Table 3) and a continuous grazing of the summer pastures west of Alesjaure, allowed reindeer to recover after the harsh winter and spring and to build new energy reserves.

### Herding season 2017–2018

AWS Mella data show that temperatures varied around freezing after the snow began to accumulate in early October 2017 (Fig. 12). Herders observe that this again resulted in ice crystals forming on the ground vegetation. They, therefore, decided to keep the reindeer west of Kiruna for winter grazing (Fig. 13). Two massive snow fall events were recorded by the weather stations in the middle of November and in early December (Fig. 12), creating an unusual snow accumulation gradient, with higher snow depths to the east and very little snow to the west of the highest mountains (i.e., around Alesjaure). The deep snow lured the reindeer towards the west to the summer pasture areas of Alesjaure and all the way into Norway (Fig. 13). The herd was scattered widely and gathering the reindeer for siida separation, therefore, became a challenging and costly operation requiring helicopter assistance. Luckily, there were enough snow-free patches with exposed lichens to just sustain the herd through the winter in the west.

The herders hoped that the snow would melt early in the calving area in Laevasvággi and, therefore, guided the reindeer back there at the end of April (Fig. 13). The remaining snowpack registered by the AWS Laevasvággi (Fig. 12) explain why the reindeer returned towards the west to Alesjaure before calving in mid-May. The reindeer remained there until the calves were strong enough for the move to Laevasvággi which was snow free at the end of May (Fig. 13). The weather data show unusually high July temperatures (Fig. 12; Table 3). As revealed by the GPS data, the heat forced the reindeer to spread to higher ground where snow fields and glaciers afforded solace to the heat and many insects. To reduce imposed stress on the animals, the herders halved the number of round-ups for calf labeling (A. Inga, pers. comm.). The herders associate the lower weight of calves and adult reindeer observed during the autumn round-ups to the combination of poor winter grazing and relatively low nutrient intake due to the summer heat.

## Discussion

Average air temperatures have increased in northern Sweden during all seasons over the past three decades (SMHI 2020). A largest increase of 2 °C occurred during the winter season (December–February). An important implication of warmer winters is that periods with positive air temperature and rain occur more frequently especially in the study area where large temperature fluctuations occur during winter (e.g., Kivinen et al. 2017; Marshall et al. 2020). Warmer winter weather has resulted in more frequent formation of very dense snow and ice layers on the ground or within the snowpack (this study; Johansson et al. 2011; Rasmus et al. 2018). It is evident from our results that even a single rain-on-snow event, or an extreme snow fall event, can adversely impact reindeer ability to find forage. The timing of such events during late autumn and early winter has a decisive impact on how grazing conditions develop later in the season. Our results show that in their wide response to such extreme weather and situations, reindeer will seek all alternative escape routes, including those that are highly dangerous. The importance of maintaining fences along roads, railroad-tracks, and other dangerous infrastructures increases.

If positioned on the lower mountain plateaus west of the major infrastructures at Kiruna reindeer may respond by retreating into the higher terrain in the west (e.g., herding seasons 2015–2016 and 2017–2018; Figs. 9, 13). Grazing there should be avoided in winter because the vegetation constitutes an important resource for spring and autumn grazing.

The reindeer response to weather and snow stress has also other important economic implications. For example, as reindeer herds become widely dispersed across the mountain plateaus, it is more demanding to keep them together and herders require a more frequent use of helicopters to locate and gather the animals (e.g., herding season 2017–2018). Likewise, the transport of trapped reindeer with trucks towards suitable pastures also increases during times of weather-imposed stress (e.g., herding season 2013–2014, Fig. 5). Both modes of transport result in increased carbon footprints and cost. In emergency situations, herders may also need to supplementary-feed reindeer which is expensive, increases the risk for infectious diseases, and has a negative impact on reindeer behavior (Tryland et al. 2019; Horstkotte et al. 2020). Laevas RHC successfully received disaster compensation (Sveriges Riksdag 1993) from the Sámi Parliament for fodder for c. 2500 animals during four of the five winters of the study period 2013–2018.

Our results show that the importance of access to alternative mature forests with arboreal lichens increases when snow and ice conditions hinder reindeer grazing of ground lichens in the pine forest. Compared to conditions that allow for grazing terricolous lichens, the reindeer need to disperse over a larger area when they have to sustain themselves on arboreal lichens. When icing also restrict accessibility of arboreal lichens, the siida cannot be sustained for very long in these types of ecosystems. Due to forestry it has become an increasingly difficult task to find mature forests suitable for winter grazing that are unoccupied by other siidas or RHCs. We note that during all the winters when reindeer were herded to the traditional winter grazing ranges at Mella (herding seasons 2013–2014; 2014–2015, 2016–2017) ice-locked pastures forced them to move to forests with arboreal lichens to survive.

One effect of high summer temperatures is that reindeer graze at high altitudes, often spending time on snowfields, where food is sparser (e.g., herding seasons 2013–2014 and 2017–2018). Herders have noted that the lower nutrient intake results in lower calf growth rates and thus also likely in a reduction in available energy reserves for the winter (Thompson and Barboza 2017). Under such circumstances the number of calf labeling round-ups at established corrals are limited or avoided entirely to alleviate stress on the reindeer. Therefore, opportunities to associate calves to the correct siida are reduced and may result in missed calf labeling, property loss, and lowered income.

Future climate scenarios for northern Sweden predict higher temperatures during all seasons and increased winter precipitation (Berglöv et al. 2015). Hence, the frequency of rain-on-snow and high snow accumulation events will most likely continue to increase, which will further increase climate stress on reindeer herding. The areal coverage of summer snow fields as refuges that provide important cooling and below which exists continuous fresh summer vegetation, is likely to continue to decline (Kivinen et al. 2012b). The impacts of increased warming and more variable weather and snow conditions on reindeer herding are not exclusive for Laevas RHC. On the contrary it is a major concern among all RHC in Sweden, and among herding communities in other Arctic regions (e.g., Löf et al. 2012; Mathiesen et al. 2013; Uboni et al. 2016). Laevas RHC, however, experiences many additional land-use changes such as infrastructure development associated with the mining industry (LKAB, Laevas- och Gabna samebyar 2015), the relocation of the township of Kiruna (Sjöholm 2013), forestry, and tourism (Fohringer et al. 2021). The number of areal and linear infrastructures has increased, and therefore reduced pasture areas and connectivity between remaining pastures over the past 130 years (Fohringer et al. 2021). Several important migration routes that used to pass Kiruna have been cut off by the expansion of mining-related infrastructure (LKAB, Laevas- och Gabna samebyar 2015). Due to forestry there has been a decline in the lichen-abundant old-growth forests within Laevas RHC grazing ranges, as it has been in northern Sweden generally (Kivinen et al. 2012a; Sandström et al. 2016). The establishment of wind farms in the winter grazing lands further increases fragmentation and the noises of windmills in operation disturb the reindeer directly (Skarin and Åhman 2014; Skarin et al. 2018). Disturbance from mountain tourism (Øian et al. 2018) has also become more of a problem in recent years. This is partly due to an increased accessibility to remote, previously undisturbed areas due to helicopter services.

For the Sámi reindeer herding culture to also flourish in the future, it is pertinent that their adaptive capacity to warmer and more variable weather and snow conditions increases. On the contrary, we observe a continued reduction of the adaptive capacity of Laevas RHC because of an increase of competing land-use activities (Fohringer et al. 2021). It is, therefore, pertinent that environmental planning efforts also integrate the effects of climate change on the availability of ecosystems for reindeer grazing. By endorsing the need for connected and undisturbed pastures in future land-use planning actions (including a variety of food sources; i.e., arboreal and terricolous lichens), by improving the situation relative to today, some of the effects of climate change on reindeer herding can potentially be mitigated. If in the modern Swedish society, its Indigenous peoples are to be supported to uphold their tradition of reindeer herding, it becomes urgent that the threat of climate change is recognized by governmental agencies.

## Conclusions

We have used weather data, GPS tracking data on reindeer, and herder knowledge to show that temporal and spatial patterns of reindeer movements are strongly determined by changes in weather and snow conditions. Increased incidence of ice- and snow-locked vegetation, due to rain-on-snow and high precipitation snow events, demands an increased reindeer herding community flexibility in guiding their reindeer herds to suitable grazing areas during winter. We conclude that air temperature, ground temperature, and precipitation during autumn and early winter have a decisive impact on subsequent temporal and spatial grazing conditions, especially the occurrence and timing of rain-on-snow events are crucial. The timing of spring snow melt and summer temperatures greatly impact the potential for the reindeer to build necessary energy reserves. We have observed high variability in these measures over the monitored 5 years (2013–2018). Together with the cumulative effects of increased pressures of alternative land-use activities, the warmer climate and its non-predictable weather derivatives seriously challenge future reindeer herding in this area. If this important traditional culture is to develop and transition into a warmer future, the need for preserved interconnected grazing areas allowing for high flexibility in the choice of grazing land needs to be recognized, endorsed, and respected. We conclude that by combining scientific measurements and herder observations, we increased the depth of knowledge of how reindeer are impacted by changes in weather and adverse snow conditions.
